# Psychological Impact of Living Kidney Donation: A Systematic Review by the EAU—YAU Kidney Transplant Working Group

**DOI:** 10.3389/ti.2023.11827

**Published:** 2023-11-24

**Authors:** Valentine Cazauvieilh, Valérie Moal, Thomas Prudhomme, Alessio Pecoraro, Alberto Piana, Riccardo Campi, Vital Hevia, Angelo Territo, Romain Boissier

**Affiliations:** ^1^ Department of Nephrology, La Conception University Hospital, Assistance Publique – Hôpitaux de Marseille, Aix-Marseille University, Marseille, France; ^2^ Department of Urology, Rangueil University Hospital, Toulouse, France; ^3^ Unit of Urological Robotic Surgery and Renal Transplantation, Careggi Hospital, University of Florence, Florence, Italy; ^4^ Department of Urology, San Luigi Gonzaga Hospital, University of Turin, Turin, Italy; ^5^ Department of Experimental and Clinical Medicine, University of Florence, Florence, Italy; ^6^ Urology Department, Hospital Universitario Ramón y Cajal, Alcalá University, Madrid, Spain; ^7^ Oncology and Renal Transplant Units, Puigvert’s Foundation, Barcelona, Spain; ^8^ Department of Urology and Renal Transplantation, La Conception University Hospital, Assistance Publique – Hôpitaux de Marseille, Aix-Marseille University, Marseille, France

**Keywords:** living kidney donors, quality of life, anxiety, depression, regret

## Abstract

We performed a systematic literature review of the psychological impact on donors of living kidney donation. We conducted a literature review in PubMed/Medline according to PRISMA guidelines which included both qualitative (based on interviews) and quantitative studies (based on standardized questionnaire). There were 15 quantitative studies and 8 qualitative studies with 2,732 donors. Given that the methodologies of qualitative and quantitative studies are fundamentally different, we narratively synthetized results of studies according to four axes: quality of life, anxiety/depression, consequences of donation on the donor/recipient relationship, overall satisfaction and regret. The quantitative studies reported that donor quality of life remained unchanged or improved. Donor regret rates were very low and donor-recipient relationships also remained unchanged or improved. Qualitative studies reported more complex donation experiences: one can regret donation and still decide to recommend it as in a social desirability bias. In both study types, donor-recipient relationships were closer but qualitative studies reported that post-donation rebonding was required. The qualitative studies therefore highlighted the psychological complexity of donation for donors, showing that living donation impacts the donor’s life whether it is successful or not. A better understanding of the impact of donation on donors could provide better care for donors.

## Introduction

Kidney transplantation is currently the best treatment for patients with end-stage renal disease [1, 2]. However, the number of available organs is too limited to meet the growing demand for transplantation. This situation has led to the development of living-donor (LD) kidney transplantation, a practice that allows for the transplantation of better-quality kidneys with a longer lifespan than grafts from deceased donors. In an increasing number of countries, living donors who present themselves as potential candidates for donation are no longer only close family members or biologically related to the recipient [3]. Living donation is at a crossroads in medicine with a greater need for organs for patients waiting for transplants. Current studies therefore deal with the psychological repercussions of such a procedure on the donor and on the psychological evaluation of him or her in the living donor process [4]. At present, recommendations do not require living donors to have a psychological evaluation before donation but it is nevertheless “strongly recommended” [5]. The increase in this activity is prompting studies to look more closely at factors that could influence the mental health of living donors.

The psychological impact of donation on donors can be assessed through two different methodologies: quantitative studies assessing the donor with tests and questionnaires as well as qualitative studies that evaluate the donor’s subjective experience assessed through research interviews. Quantitative studies based on standardized questionnaires are significantly cited when supporting organ donation since they report an increase in donor quality of life after donation compared with the pre-donation period [6]. Conversely, qualitative studies based on interviews report that donation has an impact on donor lives and that it necessarily induces a renegotiation of their identities, roles and relationships with the recipient [7, 8].

The aim of this study was to review the literature for studies of the psychological experience of donation among living donors. Contrary to previous reviews, we aimed to include both quantitative and qualitative studies in order to better understand the psychological impact of donation on the donor.

## Materials and Method

### Search Strategy

**FIGURE 1:**
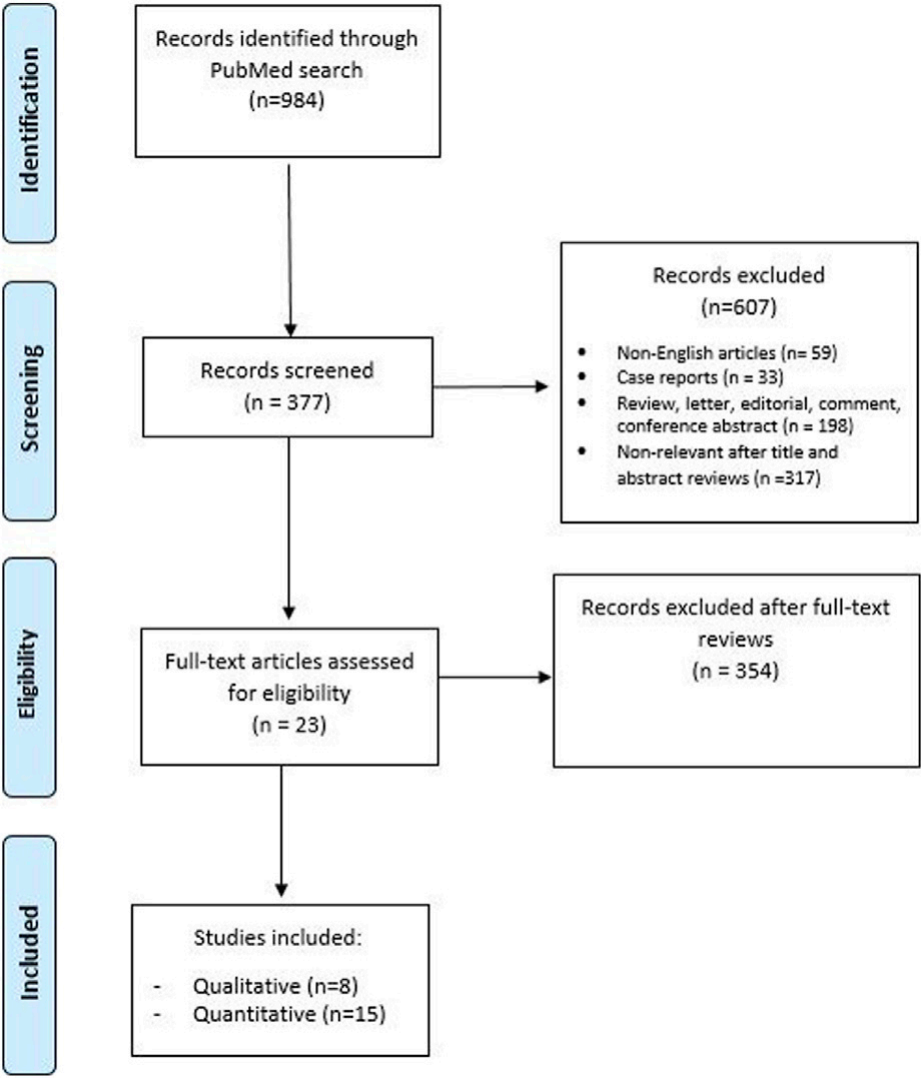
PRISMA flow chart.

We conducted a systematic review in line with Preferred Reporting Items for Systematic Reviews and Meta-analyses (PRISMA) guidelines [9] (Figure 1).

A literature search was conducted up to 31 October 2022 in PubMed/Medline. The following keywords were used in our search strategy: (renal transplantation or kidney transplantation) AND (living donor nephrectomy) AND (quality of life OR anxiety OR depression OR regret).

### Inclusion and Exclusion Criteria

We included studies which analyzed the psycho-social impact of living renal donation with standardized questionnaires or interviews. In case of duplicate publications, either the higher-quality or the most recent publication was selected. Reviews, meta-analyses, letters, editorials, meeting abstracts, author replies, case reports, and non-English articles were excluded. Studies dealing with living donor nephrectomy which did not consider the postoperative psycho-social impact of donation as a primary endpoint were excluded. Since the impact on the relationship with the donor was part of our aim, studies that included anonymous donation were excluded. No restriction on publication date was applied.

Initial screening was performed independently by two investigators based on the titles and abstracts of articles to identify ineligible reports (VC and RB). Potentially relevant reports were subjected to a full-text review and the relevance of the reports was confirmed after the data extraction process. Disagreements were resolved by consultation with a third co-author (VM).

### Data Extraction and Analysis

Two review authors (VC and RB) performed independent initial screening based on the titles and abstracts. Studies were allocated to the group “Quantitative study results” when a standardi**z**ed questionnaire was used and were allocated to the group “Qualitative study results” when the evaluation was based on interviews.

Both authors independently extracted the following variables from the included studies: first author’s name, publication year, country of research, study design, period of patient recruitment, number of patients included, type of evaluation (quantitative vs. qualitative).

### Risk of Bias Assessment

**FIGURE 2:**
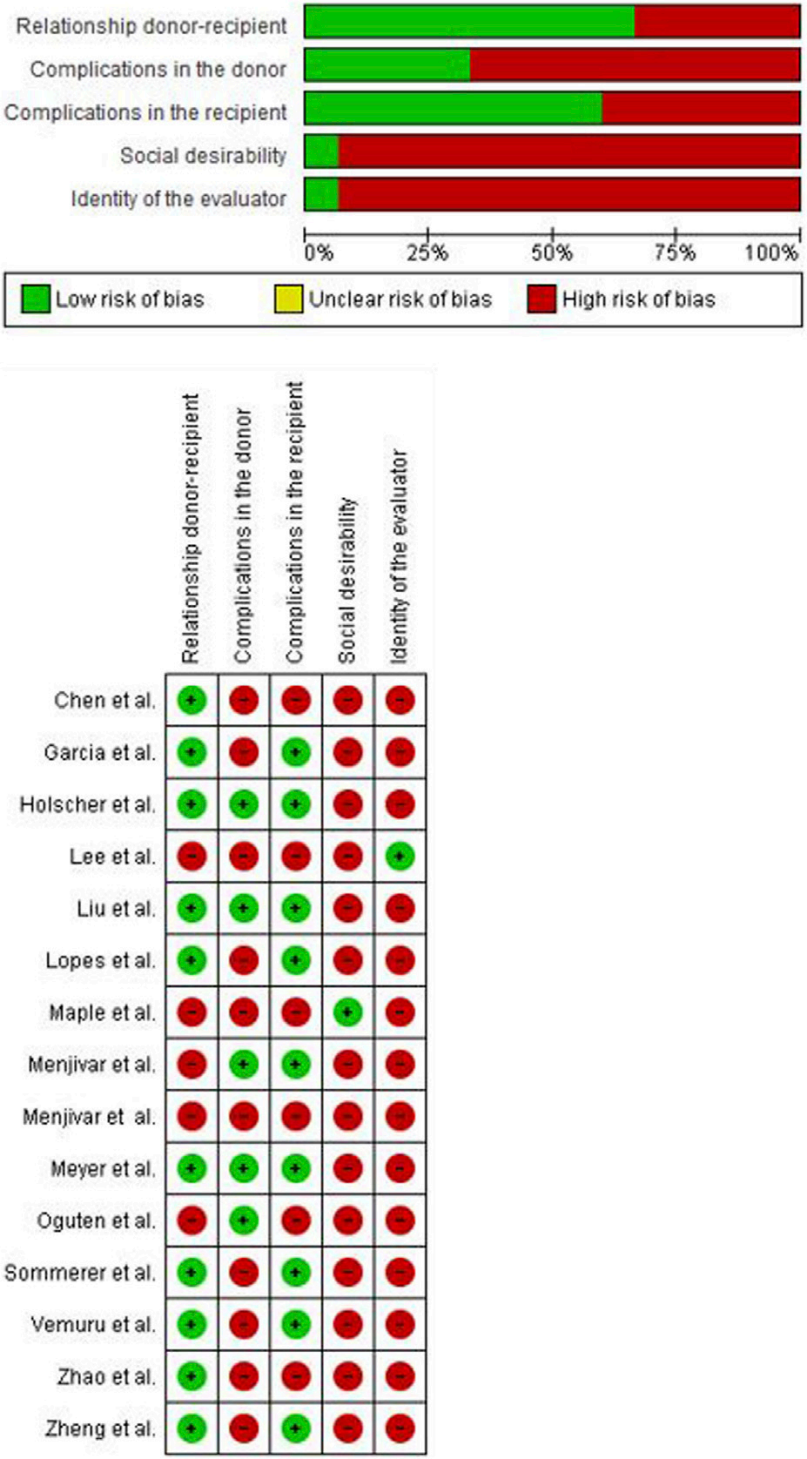
Risk of bias quantitative studies.

**FIGURE 3:**
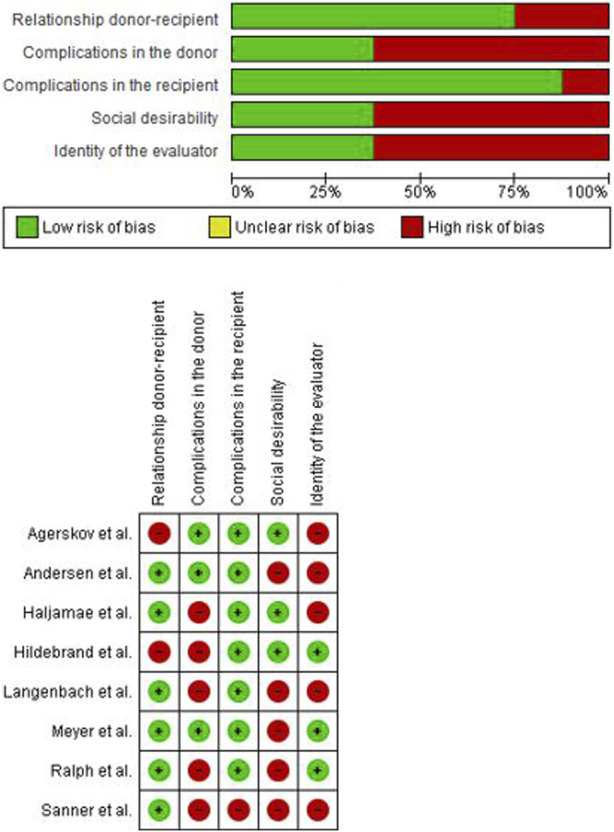
Risk of bias qualitative studies.

The risk of bias (RoB) of the included studies was evaluated according to the “Risk of Bias in Non-Randomized Studies of Interventions (ROBINS-I)” tool [10]. ROBINS-I is the recommended tool for Cochrane Reviews for non-randomi**z**ed studies of interventions. In addition, two reviewers independently assessed the RoB using five confounding factors which were identified *a priori*: donor-recipient relationship, medical/surgical complications in the donor, medical/surgical complications following renal transplantation in the recipient, social desirability, identity of the evaluator. The RoB summary and graph figures were generated using the Cochrane Review Manager 5.4 (RevMan 5.4; The Cochrane Centre, Copenhagen, Denmark). The overall RoB level was judged as “low,” “unclear,” or “high” risk (Figures 2, 3).

### Analysis

The planned meta-analysis of the psycho-social impact of living donation evaluated quantitatively by standardized questionnaire was not possible owing to the heterogeneity of the questionnaires used in the literature. Since the methodologies of qualitative and quantitative studies are fundamentally different and cannot be compared in a classical systematic review, we choose to narratively synthetize the results of both types of studies according to four axes: quality of life, anxiety/depression, consequences of donation on the donor/recipient relationship, overall satisfaction and regret.

## Results

### Donor Characteristics

Across all studies, a total of 2,732 donors were assessed. The mean age on donation was 49 years, the majority of donors were female (61%) and employed. None of the studies provided exact descriptions of the donor-recipient relationship but we can observe that the majority of them were genetically related and were parents (22.7%), siblings (19.4%) or spouses/partners (15.0%). Concerning marital status, type of the surgery, religious belief, the studies do not report enough data for analysis (Table 1).

**TABLE 1:** Demographic data of donors in all studies.

Variables	Statistic *n*, (%)
Age (y)	49
Gender (*n* = 2,732)	
Male	1,060 (38.8)
Female	1,672 (61.2)
Donor-recipient relationship (*n* = 1,899)	
Parent	431 (22.7)
Sibling	368 (19.4)
Spouse/partner	285 (15.0)
Child	48 (2.5)
Friend	39 (2.0)
Third and fourth degree	22 (1.2)
Other unspecified (related)	95 (5.0)
Genetically related (unspecified)	400 (21.1)
Emotionally related (unspecified)	211 (11.1)
Occupation (*n* = 1,785)	
Employed	1,205 (67.7)
Unemployed	325 (18.2)
Retired	240 (13.4)
Student	1 (0.01)
Other	14 (0.7)
Marital status (*n* = 1,215)	
Married/live with a partner	1,108 (91.2)
Single/Divorced/Widow	107 (8.8)
Type of Surgery (*n* = 601)	
Open nephrectomy	233 (38.8)
Laparoscopy	368 (61.2)

*n* = number of donors described in the studies. Marital Status and Type of surgery are not always reported in studies. The studies do not report the exact type of donor-recipient relationship and use the categories “Genetically related” and “Emotionally related.”

This review reports 15 articles with quantitative measures and 8 articles with qualitative measures. Fifteen studies were retrospective post-donation and 8 studies were prospective assessing donors before donation and then at 3 months and/or 6 months and/or 1 year and up to 10 years after donation (Tables 2, 3). Among the included quantitative studies, half used the Short-Form Health Survey-36 item (SF-36). Owing to the heterogeneity of the questionnaires used and the limited number of studies that used the most represented test (SF-36), a meta-analysis could not be considered.

**TABLE 2:** Characteristics of quantitative studies.

Source	Journal	No. of donors	Country	Years of inclusion	Methods (tests)	Time since donation				Psycho-social outcomes following living donation				
										Quality of life	Anxiety depression	Regret	Impact failure/death	Donor/recipient relationship
[11]	Asia Pac Psychiatry	98	Asia	2008–2010	SDS-SAS- SSRS -SF-36	5−1 y				Yes	Yes	No	No	Yes
[12]	Clin Transplant	50	Brazil	2007–2009	Donor Questionnaire—SF-36	3–12 m				Yes	No	Yes	Yes	Yes
[13]	BMC Nephrol	825	United States	2011–2017	GAD-2 - PHQ-2 - 1 question regret	3–10 y				No	Yes	Yes	Yes	No
[14]	BMC Nephrol	53	Korean	2008–2019	MMPI-2 - STAI - CES-D	—				No	Yes	No	No	No
[15]	Transplant Proc	41	Taiwan		The Decision Regret Scale - Effective decision subscale - SF-12	>3 m				Yes	Yes	Yes	No	Yes
[16]	Transplant Proc	45	Portugal	2002–2008	Socio-demo-test - SF-36	>12 m				Yes	Yes	No	No	No
[17]	BMC Nephrol	217	Norway	2013	SF-36-MFI and specific questions	8–12 y				Yes	No	Yes	Yes	Yes
[18]	Transpl Int	100	England	2012–2013	Questionnaire by the research team	3 and 12 m				Yes	Yes	Yes	Yes	Yes
[19]	Sci Rep	60	France, Germany, Portugal, Spain, Sweden	2011	ACASA - SF-36 - HADS - LOTR - SOCS - EPQ-RA - ELSA	12 m				Yes	Yes	No	Yes	Yes
[20]	BMC Nephrol	332	Spain	2005–2015	EULID - ESS	>12 m				No	No	Yes	No	No
[21]	Transplant Proc	208	Turkish	2006–2017	BDI - BAI - CLAS	1–12 y				No	Yes	Yes	No	Yes
[22]	BMC Nephrol	211	Germany	1983–2011	SF-36 - MFI-20 - PHQ-9	—				Yes	No	No	No	No
[23]	Indian J Uol	100	India	NE	WHO QoL BREF	6 m				Yes	No	No	Yes	No
[24]	BMC Nephrol	84	China	2002–2007	BDI - SAS - SSR - SF-36 – 22-item sociodemographic	6–12 m				Yes	Yes	Yes	No	Yes
[25]	Transplant Proc	110	China	2002–2012	SAS - SDS – Self-made socio-demographic questionnaire	1–106 m				Yes	Yes	Yes	No	Yes

SDS, self-rating depression scale; SAS, self-rating anxiety scale; SSRS, social support rating scale; SF-36, the short-form-36; BDI, the beck depression inventory; BAI, the beck anxiety inventory; GAD-2, the 2-item generalized anxiety disorder scale; PHQ-2, the 2-item patient health survey; PHQ-9, the patient health survey questionnaire-9; CSQ-8, the client satisfaction questionnaire; LOT, life orientation test; MMPI-2, the Minnesota multiphasic personality inventory-2; STAI, the state trait anxiety inventory; CES-D, the center for epidemiologic studies depression scale; EULID, European living donation and public health project; ESS, European social survey; ACSA, anamnestic comparative self-assessment; HADS, hospital anxiety depression scale; LOT-R, life orientation test; SOCS, sense of coherence scale; EPQ-RA, Eysenck personality questionnaire-revised-abbreviated; y, year(s); m, month(s); —, not evaluated; Yes, evaluated; No, not evaluated; NE, not evaluated.

**TABLE 3:** Characteristics of qualitative studies.

Source	Journal	No. of donors	Country	Years of inclusion	Methods	Time since donation
[26]	J Rend Care	18	Denmark	2012–2013	Interview−observation	1 week before donation (do); 3 months after do
[27]	Clin Transplant	12	Norway	2004	Semi-structured telephone interview	1 year after do
[28]	Clin Transplant	10	Sweden	1997	Interview	>3 years after do
[29]	Clin Transplant	76	United States	NE	Telephone survey−interview	1–6 years after do
[30]	Transplant Proc	11	Germany	NE	Semi-structured interview	2–3 years after do
[31]	BMJ Open	16	Norway	2014–2015	Semi-structured interview	>10 years after do
[32]	BMJ Open	16	Australia	2014–2017	Face-to-face semi-structured interview; telephone	Before & 11–14 m after do
[33]	Nephrol Dial Transplant	39	Sweden	2000	Open interview	1 day before−3 weeks after do

### Narrative Synthesis of Evidence

#### Quality of Life

##### Quantitative Study Results

The concept of quality of life is one of the main concepts used in the evaluation of the psychological and physical impact of donation on a living donor. Studies use quality-of-life measures by assessing it either prospectively or retrospectively. Prospectively, they compare donor outcomes with those of recipients, with general population norms, or compare different types of donors with one another (e.g., type of donor-recipient relationship).

The studies report two types of results. The first finding is that there is no significant difference in quality-of-life scores between pre-donation and 1 year post-donation [16, 18, 25]. The second finding is that some studies observe an increase in quality of life as early as 1 year post-donation compared with the pre-donation period [12, 22–24]. Studies also point to risk factors associated with decreased donor quality of life such as donor fatigue, anxiety depression, lack of social support, the nature of the donor-recipient bond and postoperative complications or recipient graft loss [11, 16, 17, 22, 23] (Supplementary Table S1). Socio-demographic data does not impact the quality of life [13, 18, 23]. The studies are not in agreement regarding the impact of transplant failure on quality of life [12, 23] (Supplementary Table S1).

##### Qualitative Study Results

None of the qualitative studies evaluated the concept of quality of life in their results (Table 3).

#### Anxiety/Depression

##### Quantitative Study Results

Several measures are used to assess anxiety and depression (Table 2). After donation, studies generally report a low prevalence of anxiety and depression in donors [13, 18, 21, 24, 25]. However, they also point certain risk factors associated with an increase in symptoms of depression and anxiety. Donors who experienced postoperative complications or recipient graft loss had more anxiety and lower life satisfaction [13, 18, 21]. There was an “emotional contagion” [14] from recipient to donor meaning that recipient anxiety or depression could impact the donor. Chen et al. reported that parent donors showed more anxiety and depression than sibling donors [11] but another study found no impact of the nature of relationship on the donor [21] (Supplementary Table S1). Education, marital status and gender also appeared to be risk factors [13, 22].

##### Qualitative Study Results

The studies noted that the majority of donors reported post-donation depression and anxiety and also great difficulty adjusting after nephrectomy with aggression, hyper-vigilance about wellbeing and fear of rejection by the recipient [26, 27, 30, 32, 33]. The donors also reported feelings of vulnerability associated with intense fatigue [26, 27, 31, 33] after donation. They explained this as a result of having to go from a healthy person to someone who has undergone surgery [26]. It was also disappointed expectations that impacted the donation experience [26, 30, 32] as well as the evolution of the donor-recipient relationship or failure of the transplant or the death of the recipient [27, 28, 32]. One study found that the donation event took a back seat with the passing of time [31].

Study results were diverse concerning the psychological impact on donors vis-à-vis complications, transplant failure or recipient death. Some studies reported that donors experienced feelings of guilt such as not having donated a good enough kidney, grief, depression, a sense of fault and responsibility, disappointment, severe psychological distress, and physical symptoms [27, 28]. Conversely, another study reported that some donors denied feeling guilt or regret over the failure of the transplant because they felt they had done the right thing for their families [27]. Over time, donors appeared to have accepted the failure of the transplant as their fate or in a less negative manner [27, 28].

Despite the obviousness of the decision [28, 29, 31–33], donors explained the dilemmas, ambivalence and anxiety they faced [26, 32]. Donor dilemmas were sometimes reinforced by close friends or family who showed “overwhelming concern for the donor’s health” or questioned the donor’s decision. The pre-donation evaluation period is often very distressing for donors [26, 32, 33].

#### Consequences of Donation on the Donor/Recipient Relationship

##### Quantitative Study Results

The quality of the donor-recipient relationship was assessed using specific questions about improvement of the relationship after donation (Table 2). The majority of studies reported that the relationship of the donor-recipient dyad remained either unchanged [19, 21] or had improved and become “closer” [12, 21, 24, 25]. However, there were a few cases where the relationship deteriorated after donation [17–19, 21, 25] (Supplementary Table S1).

##### Qualitative Study Results

In general, the studies found that the relationship between donors and recipients either remained unchanged or a special bond developed between them. Donors reported a closer and stronger bond, a better understanding of each other, a more dynamic dyad, and a more balanced relationship [26, 27, 29, 32]. However, this closer donor-recipient bond was not always the case. This relationship was associated in many dyads with a renegotiation of roles, expectations of the recipient, conflict, tension owing to disappointment, unmet expectations, broken contact or divorce after donation [27, 29, 31, 32]. The nature of the donor-recipient relationship appeared to impact the type of motivation and secondary benefits expected from the transplant [28, 31–33].

Donor psychological experience appeared to be dependent on the realization of initial expectations and expected benefits from the transplant. The donors who achieved a personal benefit after donation related to the success of the transplant participated in a positive donation experience [27, 32]. Donors reported difficulty in having to fill multiple roles after donation. For parents, there was an increase in tension and stress with other family members or it was difficult for some to balance work and family responsibilities [27]. Some donors hoped that the caregiver role would diminish after donation, especially in the case of spousal dyads. When this did not happen, donors felt disappointed or frustrated [32]. Donors expressed negative donation experiences when recipients were not compliant [27, 29, 32]. The studies showed that the living donor transplant process involved not only the recipient and his or her donor but the entire family [31, 33]. As for the impact of transplant failure on the donor, there was no impact on the donor-recipient relationship [30].

#### Overall Satisfaction and Regret of the Donation

##### Quantitative Study Results

The studies were in agreement that in the majority of cases, donors were satisfied with the donation process and remained committed to their decision [12, 15, 20, 21, 25]. The vast majority of donors did not regret the donation [12, 13, 17, 18, 20, 21, 23–25]. Several studies attempted to quantify donation regret with specific questions such as “If you had to do it again, would you?” “Would you recommend it?”. The results of the studies showed that it is possible to regret the donation and still recommend it or agree to do it again if possible and *vice versa* [15, 20, 24, 25] (Supplementary Table S1).

All of the studies reported a low rate of regret in donors at different times after donation. The authors reported correlations between regret and fatigue rates [17], regret and graft failure or complications in the recipient [17], while other studies found no correlation between donor regret and recipient complications or death [12, 15, 19]. There was also a correlation between donor regret and deterioration of the relationship with the recipient after donation as well as a correlation between donor regret and anxiety and depression [13, 21]. On the other hand, the percentages in the studies showed that the majority of donors expressed that they would donate again if they had to but there was also a decrease in percentages when the donor was asked if he or she “would encourage donation” [15, 24, 25] (Supplementary Table S1).

##### Qualitative Study Results

Donors reported an ambivalent donation experience [26, 33]. Most of them were very satisfied with the donation and the positive effects observed in the recipient [26, 27, 29, 30]. This feeling was reinforced by the family, social environment, the recipient and sometimes the transplant team, who made them proud of their gesture [27, 29, 31–33]. However, the donation experience was still described as an “overwhelming experience” [26] that was not always positive and was often marked by a feeling of vulnerability in the donor after surgery [26, 27, 29, 30, 32, 33]. The donors who were the least supportive of donation were those who experienced transplant failure in the recipient [29]. However, in the majority of cases, even the least supportive donors reported that they would donate again if necessary [29].

#### Subjects Added to the Analysis of the Qualitative Studies

The qualitative studies reported other findings that focused on the pre-donation period that appeared to be necessary for outcome analysis.

##### The Pre-Donation Period

The pre-donation evaluation period is often not a good experience for the donor [26, 32, 33]. Some find it “the worst step” in the donation process. It is a very anxious and uncertain period where the donor is confronted with both his or her own dilemmas, the fear of being rejected as a donor, the long wait for test results, etc. During this period, donors also reported a feeling of being “out of touch” with their families with feelings of abandonment and loss.

##### The Use of Strategy to Influence the Transplant Team

Several studies reported that some donors used strategies to try to influence transplant teams to select them as donors [26, 29, 33]. These donors tried to convince teams that they were not psychologically fragile, that previous psychological problems would not interfere with donation, and that their physical health was not a barrier. For some, the explanations given concerning their motivation were thought out beforehand so they would not be misinterpreted. Some donors withheld information from the transplant team to increase their chances of being selected as a donor [29].

##### Sense of Abandonment and Support

Some donors reported feeling forgotten, lost and abandoned after donation whereas they were considered “sensational” prior to donation [27, 30, 33]. They expressed the importance of medical follow-up after donation in order to feel supported and reassured [27, 29, 31, 33]. They also criticized the lack of active approach by transplant teams during post-donation follow-up, especially when the transplant failed or the recipient died [27, 28, 31].

## Discussion

Our initial aim was to review recent literature in order to better understand the psychological impact of living donation on the donor in renal transplantation. Living donation is currently the most favorable solution for patients waiting for a kidney transplant. This literature review focused on quantitative and qualitative studies. At first glance, it is not intuitive. However, this is also what gives it a unique approach. We thought it would be of interest to place the results of two very different study methods side by side in the same article. The results are very different and give rise to new questions about living donation.

Quantitative studies have reported that quality of life, anxiety, and depression in donors remained unchanged after donation whereas in prospective studies quality of life improved after donation. The rate of regret among donors was very low and the donor-recipient relationship also remained unchanged or improved after donation. Qualitative studies reported a more complex donation experience that included positive experiences, vulnerability, ambivalence and anxiety. Relationships with the recipient were closer as shown in the quantitative studies but they had to go through a post-donation reshaping of relationships, a renegotiation of roles and expectations.

Analysis of the qualitative literature revealed that the concepts used are not the same as in the analysis of the quantitative literature. Indeed, the method used with semi-structured research interviews allowed donors to disclose their experiences which generated free-flowing and unanticipated commentary, whereas the quantitative studies evaluated precise and predefined concepts based on scales and questionnaires.

The qualitative studies showed that the donors used conscious or unconscious strategies to influence the transplant team to select them as a donor during the pre-donation period. Indeed, they wanted to prove that they were in good mental and physical health and therefore fit to donate their kidney. In this light, the results of the quantitative studies are questionable and comparison of the results of the pre-donation and post-donation periods would appear to be difficult to interpret. In addition, the majority of studies did not take into account the impact of social desirability bias on the results obtained. This bias represents the tendency of individuals to give socially desirable answers when responding to surveys or personality tests [34]. It is a bias that influences the responses to questionnaires or tests administered. The donor answers what he or she thinks is expected and does not want to give answers that would make him or her look bad. In this type of study, it is conceivable that the answers given by the donor evaluated in the pre-donation or post-donation period could be influenced by this bias [18, 28, 35].

This literature review highlighted the difficulty in assessing donor regret after donation. Indeed, the results and scores showed that it is possible to regret the donation and still decide to donate again if possible or to recommend it. The qualitative studies also affirmed that a negative donation experience does not necessarily undermine the donor’s decision. The answer to the question “Would you donate again if possible?” does not guarantee whether or not the donor regrets the decision. The concept of regret seems to be much more complex. This is more especially the case if we take into account the impact of social desirability. It appears to be difficult to know whether donors are able to consciously assume regret in this evaluation process or in their lives.

As for the relationship between the donor and the recipient, the quantitative studies showed that overall donation had no impact on the donor-recipient relationship or that the relationship was often “closer.” The qualitative studies reported the same result. However, the relationship and roles often needed to be reshaped [8, 36, 37]. Even if the donor reported that the donation did not have an impact on his or her relationship with the recipient, his or her account suggested that the donation was a very present event in the relationship [7]. The Marcel Mauss theory teaches us that one gift always awaits another: the counter-gift [38]. This is the basis of a social bond. The gift necessarily implies in the relationship to the other the question of symbolic debt and guilt [39, 40].

Quantitative and qualitative studies pay little attention to the psychological consequences on the donor if post-donation surgical complications occur. They are more interested in the consequences that the recipient’s complications have on the donor. Donors are very motivated by the idea of helping a family member or a sick relative but at the same time, they feel doubts and fears about the operation that will affect the integrity of their body. Our study reports donors with different cultural background and socio-demographic data (Tables 2, 3).

According to previous studies, healthy donors undergoing nephrectomy are subject to stress events that they must adapt to [36, 41, 42]. This ambivalence towards donation does not in any way call living donation into question. On the contrary, it makes it possible to understand and therefore to accompany in a more precise manner the experience of donors which oscillates between an almost unanimously positive experience and feelings of vulnerability, anxiety, disillusionment and doubt. The results do not call into question the merits of living donation but do allow us to consider donation from another angle. It is no longer a question of identifying the impact of donation on the donor in terms of a positive or negative experience but rather as a singular experience where the donor must co-construct his or her desire to donate, his or her expectations, the reshaping of social and family relationships, the relationship with his or her body. These studies highlighted the importance of the psychological support needed before, during and after the process.

## Conclusion

In conclusion, this review of the literature showed complementary and sometimes conflicting results between quantitative and qualitative studies. The quantitative studies reported that donor quality of life remained unchanged or improved while this point was poorly evaluated in qualitative studies. Donor regret rates were very low and donor-recipient relationships also remained unchanged or improved. Qualitative studies reported more complex donation experiences, showing it is possible to regret donation but still decide to repeat or recommend it. The concept of social desirability could bias the analysis of psychological outcomes in the donor. In view of these results, it would appear important to remember that living donation has an impact on the donor’s life as soon as he or she engages in this type of procedure whether it is successful or not. Qualitative results may be useful to shape future quantitative studies and to interpret past ones. The transplantation team and the psychologist must accompany the donor to reflect on his or her decision to donate, even if it carries implicit constraints, so that the donor is a player in his or her decision.

## Abbreviations

QoL, quality of life; LV, living donor; SDS, self-rating depression scale; SAS, self-rating anxiety scale; SSRS, social support rating scale; SF-36, the short-form-36; BDI, the Beck depression inventory; BAI, the Beck anxiety inventory; GAD-2, The 2-item generalized anxiety disorder scale; PHQ-2, the 2-item patient health survey; PHQ-9, the patient health survey questionnaire-9; CSQ-8, the client satisfaction questionnaire; LOT, life orientation test; MMPI-2, the Minnesota multiphasic personality inventory-2; STAI, the state trait anxiety inventory; CES-D, the center for epidemiologic studies depression scale; EULID, European living donation and public health project; ESS, European social survey; ACSA, anamnestic comparative self-assessment; HADS, hospital anxiety depression scale; LOT-R, life orientation test; SOCS, sense of coherence scale; EPQ-RA, Eysenck personality questionnaire-revised-abbreviated.
