# Supplementary material for: Psychological Impact of Living Kidney Donation: A Systematic Review by the EAU—YAU Kidney Transplant Working Group
Source: Transpl Int. 2023 Nov 24;36:11827. doi: 10.3389/ti.2023.11827 (PMC10703979; doi:10.3389/ti.2023.11827)
Supplement: Supplementary file 1 [file Table1.docx]

| **Supplementary Materials Table 1: Synthesis of the key messages in quantitative studies** | | | | |
| --- | --- | --- | --- | --- |
| **Sources** | **Quality of life** | **Anxiety and depression** | **Donor-recipient relationship** | **Overall satisfaction & donation regret** |
| Chen et al., 2015 | Lower quality-of-life scores for LD parents compared with LD siblings | LD parents presented more symptoms of anxiety and depression than LD siblings | NE | NE |
| Garcia et al., 2012 | Decrease in quality of life at 3 months post-donation. Improvement at 1 year  No impact of graft loss or death of the recipient | NE | 95% of donors reported feeling closer to the recipient after donation | The majority did not regret the donation and reported that the donation had not had a negative impact on their quality of life |
| Holscher et al., 2018 | NE | Low prevalence of anxiety and depression 6 years post-donation 4.2% had positive depression scores 5.5% had positive anxiety scores | NE | 2.1% regretted the donation correlation between regret and anxiety and depression |
| Lee et al., 2020 | NE | Correlation between anxiety and depression in the recipient and the LD-"emotional contagion" | Correlation between emotionally-related recipients with donors and anxiety | NE |
| Liu et al., 2018 | NE | NE | NE | 97.6% agreed that the decision to donate was the right one 95.1% would make the same decision again 2.4% reported regretting the donation − 7.3% did not answer the question 4.8% considered that the donation was harmful to them |
| Lopes et al., 2013 | No difference in quality-of-life scores before and 1 year after donation | Anxiety and depression are factors associated with decreased quality of life after donation | NE | NE |
| Meyer et al., 2016 | Quality-of-life scores were lower for women than for men in some domains of the SF-36, 10 y after donation | NE | Deterioration of the donor-recipient relationship impacted donor decision to donate if they were to do it again | 94% would give their kidney again if necessary 3.2% would not give their kidney again if necessary  LD who regretted giving have higher scores on the MFI Correlation between LD regret and recipient death |
| Maple et al., 2017 | No difference in quality-of-life scores before and 1 year after donation | Low prevalence of anxiety and depression and no difference at 3 time points (before, 3 months, 1 year) | In 2 cases, the relationship deteriorated after the donation | At 3 months post-donation: the regret rate was 6.8%, at 1 year 10.7%, at 1 year 10.7%. |
| Menjivar et al., 2020 | Reduced quality of life in 3 SF-36 domains at 1 year ("bodily pain" "role physical" and "vitality") | NE | Relationship between donor and recipient unchanged in most cases 1 case where the relationship deteriorated | NE |
| Menjivar et al., 2018 | NE | NE | NE | 2.38% regretted the donation during hospitalization 2.1% would not donate their kidney 1.2% would not recommend living donation All LD satisfied with the donation, recommend it and would do it again |
| Oguten et al., 2019 | NE | Low prevalence of anxiety and depression, similar to the general population | 64.4%: the relationship was unchanged  32.5%: the relationship was improved 2.4%: relationship deteriorated  No psychological impact of the nature of the relationship (biological or not) on the LD | 2.4% regretted the decision and this was associated with a high rate of depression 99.5% of DV recommended living organ donation |
| Sommerer et al., 2018 | Increase in QoL from pre-donation to 1-year post-donation  Low MCS SF-36 score correlated with high MFI-20 fatigue score | NE | NE | NE |
| Vemuru et al., 2011 | Increase in QoL from pre-donation to 1-year post-donation  Impact of graft failure or death of the recipient | NE | NE | NE |
| Zhao et al., 2010 | Increase in QoL from pre-donation to 1-year post-donation | Low prevalence of anxiety and depression  7.1% had moderate depression | 63.1%: the relationship improved and became closer | No donors regretted their decision 97.6% strongly encourage donation and would do it again if necessary |
| Zheng et al., 2014 | No difference in quality-of-life scores before and 1 year after donation | Low prevalence of anxiety and depression, score within the norm but higher than the general Chinese population | Relationship improved in most cases  5.5% reported that the donation disrupted their marriage | No donors regretted their decision 66.4% strongly encourage donation |
| *LD: living donor; QoL: quality of life; NE: not evaluated* | | | | |
